# Effects of a culturally tailored intervention on medication adherence in Chinese and Vietnamese Americans with hepatitis B: a randomized controlled trial

**DOI:** 10.3389/fpubh.2026.1681835

**Published:** 2026-03-31

**Authors:** Lin Zhu, Wenyue Lu, Zhiqing Elaine Liu, Di Zhu, Ming-Chin Yeh, Minhhuyen T. Nguyen, Yin Tan, Min Qi Wang, Grace X. Ma

**Affiliations:** 1 Center for Asian Health, Department of Population Health Sciences, Lewis Katz School of Medicine, Temple University, Philadelphia, PA, United States; 2 Department of Nutrition and Public Health, Hunter College, City University of New York, New York, NY, United States; 3 Department of Medicine, Fox Chase Cancer Center, Temple University Health System, Philadelphia, PA, United States; 4 Department of Behavioral and Community Health, School of Public Health, University of Maryland, College Park, MD, United States

**Keywords:** community-based participatory research, health behavior change, medication adherence, mHealth, virtual patient education

## Abstract

**Introduction:**

Asian Americans are disproportionately affected by chronic hepatitis B (CHB), which is caused by infection with hepatitis B virus (HBV). While adherence to antiviral medication is an effective practical approach to managing CHB and preventing liver cancer, medication adherence rates among Chinese and Vietnamese Americans with CHB, two vulnerable yet understudied populations, remain largely unknown.

**Methods:**

We designed and implemented a randomized controlled clinical trial to investigate the potential improvement of long-term adherence to HBV medication in Asian American populations. Eligible Asian American HBV patients were recruited from the Greater Philadelphia and New York City. HBV medication adherence was assessed using the 8-Item Morisky Medication Adherence Scale. We conducted ordinary least squares (OLS) regression to examine the intervention effects on medication adherence among 129 Chinese and Vietnamese Americans taking CHB medication.

**Results:**

Among 129 participants (91 Chinese and 38 Vietnamese), about three-quarters (74.4%) reported limited English proficiency. Almost one out of 10 (9.3%) did not have any health insurance. OLS regression results indicated that the intervention had a significant impact on improving medication adherence at 12-month follow-up assessment (coefficient = 0.56, *p* = 0.04). In addition, we found that depression score at baseline was negatively associated with medication adherence at 12-month follow-up assessment (coefficient = −0.10, *p* = 0.003), with other covariates held constant.

**Discussion:**

The findings show that a community-based culturally appropriate intervention significantly improved adherence to medication among Chinese and Vietnamese Americans with CHB in a 12-month period. Providing mental health support to CHB patients in this population may play an important role in improving medication adherence.

**Clinical trial registration:**

[https://clinicaltrials.gov/study], identifier [NCT04082338].

## 1 Introduction

Hepatocellular carcinoma (HCC), an important cause of which is long-term infection with hepatitis B virus (HBV) (1, 2), continues to be the most common liver cancer and the primary cause of cancer-related deaths worldwide and in the United States (3). Asian Americans are disproportionately affected by HCC (4–6). In 2023, there were 1,769 deaths due to HBV-related deaths, and those deaths among Asian Americans were 8.5 times as high as non-Hispanic White (7).

Chronic HBV infection progresses to cirrhosis in up to 40% of untreated patients, and there is an associated risk of decompensated cirrhosis and HCC (8). Proper medication adherence to chronic hepatitis B (CHB) infection plays a significant role in reducing incidence and mortality of HCC (4, 9, 10). Antiviral treatments are effective in treating CHB and in preventing the development of HCC (11, 12). Oral antiviral treatments, particularly nucleoside/nucleotide analogs, are better tolerated by patients than interferons (11, 13). Oral HBV medication includes tenofovir, telbivudine, adefovir entecavir, and lamivudine (14, 15). Entecavir and tenofovir are the preferred agents of treatment compared to other nucleoside/nucleotide analogs, which have higher rates of resistance (11).

In the past two decades, several intervention studies have focused on enhancing adherence to clinical follow-up and laboratory monitoring as a means of reducing HCC burden in Asian Americans with CHB (16, 17). Few studies, by comparison, have focused on improving medication adherence among Asian Americans with CHB. Previous research on medication adherence in HBV patients has been mostly epidemiological, assessing overall adherence level and identifying facilitators and barriers to optimal adherence behaviors. For example, in a previous study of 165 Chinese and Vietnamese Americans living with CHB, researchers found that knowledge about HBV and having lived in the US for more than 10 years were significantly associated with better antiviral medication adherence (18). In another study of 369 CHB patients in Wuhan, China, researchers found that social support, urban residency, and non-cirrhotic status were linked to better adherence to CHB medication. A systematic review and meta-analysis on CHB medication adherence among the general population found that knowledge of CHB medication, perceived benefit of treatment, remembering to take medication, and changes to daily routine were significant predictors of CHB medication adherence (19).

While intervention efforts to directly improve CHB medication adherence among Asian American patients are scarce, research suggests that several strategies could be potentially effective. Such strategies include community-based education and awareness campaigns, culturally competent patient navigations, de-stigmatization of hepatitis B, and social support (19–21). However, few intervention efforts have been implemented to test the efficacy of these components on medication adherence of Asian Americans with CHB, particularly within culturally tailored, community-based intervention settings.

To address this gap, we designed and implemented a culturally tailored, multicomponent intervention. Through a randomized controlled trial, we sought to determine whether this intervention would effectively increase long-term adherence to CHB monitoring guidelines in two Asian American groups––Chinese and Vietnamese––in the greater Philadelphia and New York City.

## 2 Methods

### 2.1 Intervention development and implementation

We developed and implemented a culturally tailored, multicomponent intervention to promote CHB medication adherence and management among underserved Asian American CHB patients in the greater Philadelphia and New York City. We used social cognitive theory (22, 23) and social-ecological perspective (24) and adopted the community-based participatory research (CBPR) approach (25) to guide the design and implementation of this intervention. We actively engaged essential stakeholders, including community-based organization (CBO) leaders, healthcare providers, and CHB patient advocates in all stages of intervention design and implementation. Through in-person meetings and teleconferences, these key stakeholders played significant roles in identifying multilevel barriers of CHB management in Asian American communities, assessing the language and cultural appropriateness of the intervention materials, improving feasibility and accessibility of the intervention, consulting on the data collection and program evaluation efforts, as well as providing input on the logistics of intervention delivery. Although the COVID-19 pandemic created significant challenges, the input and support of CBO leaders, clinical partners, and patient advocates enabled the successful completion of the study.

With the collaborative efforts of the key stakeholders and the study team, we developed a multicomponent culturally tailored intervention that consisted of three components: Virtual Patient Education (VPE), Virtual Patient Navigation (VPN), and mobile health (mHealth) text messages. VPE and VPN were delivered to the intervention group, while mHealth text messages were provided to participants in both study arms. The VPE materials, VPN procedure, and mHealth contents included key facts and hot topics of HBV management in Asian culture, which were identified and suggested by our clinical partners, CBO leaders, and patient representatives. For example, since family is a significant cultural value of Chinese and Vietnamese Americans, topics such as protecting family against CHB (in VPE materials) and family support in promoting CHB medication adherence (in VPN) were included. All the intervention materials were developed in English and translated into Chinese and Vietnamese by bilingual community health educators to make the intervention contents culturally appropriate. To ensure translation accuracy, the translated materials were proofread and certified by United Language Services, a professional interpretation and translation agent. Moreover, we consulted CBO leaders and patient representatives on the reading levels and cultural appropriateness of the translated materials to ensure they were accessible.

**Figure 1:**
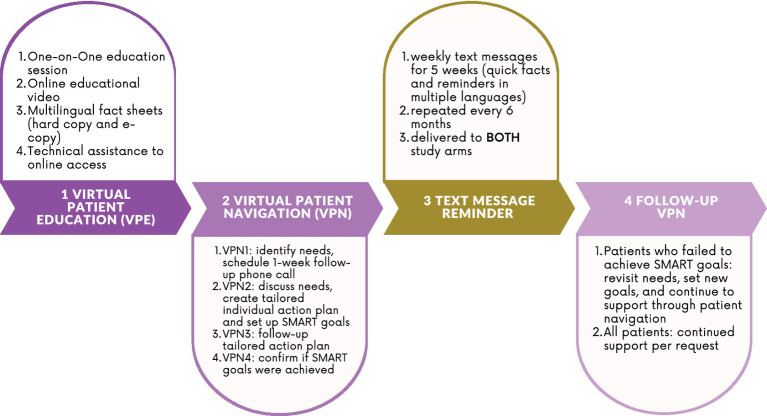
Multicomponent intervention flow chart.

Figure 1 illustrates the multiple components of the intervention. The details of each component were described in a previous study (17). In this paper, we provided a brief description of each component in the section below.

*Virtual Patient Education (VPE)*. We implemented VPE to encourage CHB management and medication adherence. The educational contents were delivered to the participants in the intervention group in three formats. First, we presented the culturally tailored CHB management and medication information in PowerPoint slides. The educational curriculum included six models: the importance of CHB management, treatment monitoring, CHB-related anxiety management, improving liver health in daily life, financial resources to access care, and family protection against CHB. Second, we provided fact sheets that included CHB facts, key epidemiological statistics, and CHB managing tips and resources. Third, we produced and presented an educational video in which two Asian CHB patients share their experiences and challenges in fighting CHB and liver cancer. We delivered VPE in two approaches. First, our bilingual Asian community health educators scheduled and conducted an in-person, one-on-one educational session with each participant in the intervention group. An electronic tablet was used to display presentation slides, fact sheets, and educational videos virtually. Once the one-on-one session was completed, the community educators shared with every participant the link to an online educational portal (http://cah-hbvtraining.net/Pages/Login.aspx) and a unique username and password to access all VPE materials on the portal. This would allow the participants to conduct self-learning and to refresh their memories on CHB management and medication adherence whenever needed.

*Virtual Patient Navigation (VPN).* To help participants in the intervention group navigate through the complex healthcare system to access CHB care, we provided a personalized one-on-one VPN. Specifically, we delivered this intervention component in four steps. In the first step, our bilingual community health navigators assisted participants in identifying barriers to CHB management and corresponding VPN needs at the initial encounter. These needs generally fell into the following categories: medication adherence support, appointment-making assistance, transportation and other logistical support, health insurance support, language support, and other specific support needs. In this initial step, bilingual patient navigators worked to build rapport and establish trust with participants, providing the foundation for future interactions. In the second step, patient navigators customized an action plan for each individual. This process was guided by the SMART (Specific, Measurable, Attainable, Realistic, and Time-bound) framework, where the patient navigator initiated a phone call with the participant within 1 week after the previous step in order to collaborate further in developing an individualized, 6-week action plan to address the identified need(s). During the interactive process, a SMART goal was set based on full agreement and understanding from the participants. In the third step, the patient navigators continued to provide navigation support via phone calls. Specifically, they made inquiries on participants’ access to care, helped them adjust their plans and goals, and provided assistance in various forms. This process was dynamic, and the action plans were adjusted based on participants’ needs and external circumstances. New challenges or barriers, such as loss of health insurance or the COVID-19 pandemic lockdowns, were discussed in a timely fashion, and solutions were found. In the fourth step, patient navigators confirmed that the SMART goals were achieved during the patient-navigator-initiated 6-week follow-up phone call. They marked the VPN component as “successfully delivered” if the SMART goals were achieved. If the participant failed to reach their goals, the navigators repeated the VPN session to address participants’ barriers to CHB management and treatment.

*Mobile health (mHealth) text messages.* There has been accumulating evidence showing that mHealth text messages are an effective tool in promoting treatment adherence among Asian Americans living with CHB (16). With the collective efforts of CBO leaders, healthcare leaders, and patient representatives, the study team developed five text messages that consisted of key information on CHB and liver damage, the importance of CHB medication adherence, and CHB follow-up appointment reminders. Participants in both the intervention and the control group received one text message each week for 5 weeks. This 5-week component was repeated every 6 months until the study was completed. Both study arms received the same text messages at the same frequency.

### 2.2 Participant recruitment

To test the effectiveness of the intervention, a two-arm randomized controlled trial was conducted from 2019 to 2021. We recruited participants through two approaches: existing cohort and CBPR recruitment, both of which were implemented with identical eligibility criteria, randomization method, intervention content, and outcome assessments. The existing cohort included 532 Asian Americans with CHB from a community-based single-blinded randomized controlled trial (20). We recruited 240 participants from this existing cohort, including 99 on CHB medication at baseline. The arm assignment of the participants recruited from the existing cohort remained the same in the present study. The second recruitment approach was CBPR recruitment, in which we recruited 142 participants (including 60 who were taking CHB medication) from 10 Asian CBOs and 5 healthcare providers. During CBPR recruitment, participants were eligible to participate if they: (1) identified as being of Chinese or Vietnamese descent, (2) were at least 18 years old, (3) were accessible by cell phone, (4) had been diagnosed with CHB for at least 12 months, (5) had not been compliant with CHB monitoring and treatment for at least 6 months, and (6) were not enrolled in other CHB management interventions. Individual-level block randomization of the 142 participants was conducted by an independent biostatistician, with the block size being 10 to ensure balance within each stratum.

**Figure 2:**
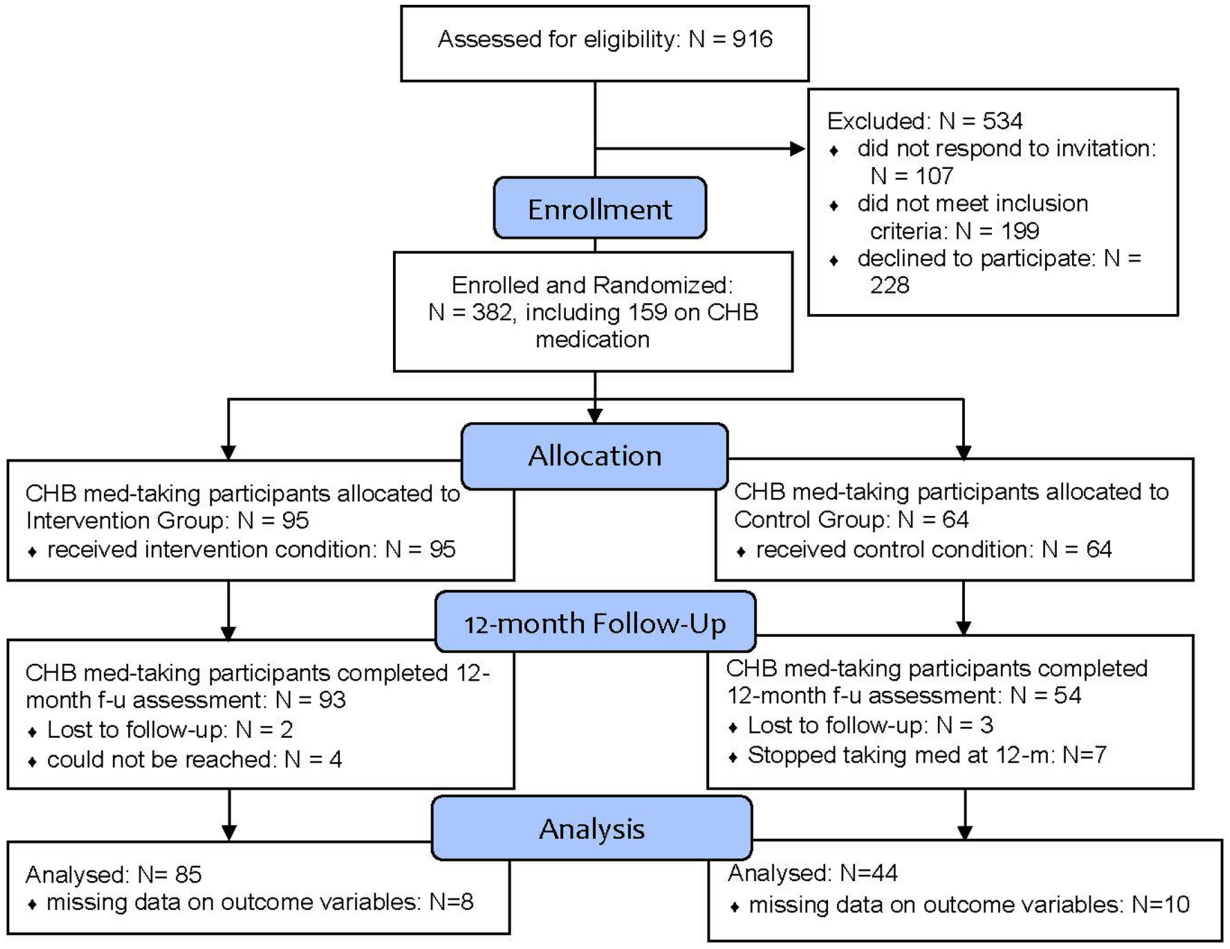
Consolidated standards of reporting trials (CONSORT) flow diagram.

We reached out to 916 potential participants, of whom 610 were eligible to participate. A total of 382 were recruited, of which 159 were taking CHB medication. Among the 159 participants on CHB medication, 95 were allocated to the intervention group, and 64 were assigned to the control group. At the 12-month assessment point, 147 participants were taking CHB medication (intervention group: *n* = 93; control group: *n* = 54), 5 dropped out of the study, and 7 stopped taking CHB medication by the 12-month follow-up timepoint. Because medication adherence was the primary outcome, and MMAS-8 scores could not be meaningfully derived for these cases, a complete-case analytic approach was used. Missing outcome data were primarily attributable to incomplete survey responses rather than study arm assignment or observed baseline characteristics. In total, we excluded 18 cases with missing values on the outcome variable at baseline and/or 12-month assessment points, leading to a total sample size of 129 (intervention group: *n* = 85; control group: *n* = 44) (Figure 2). All participants received informed consent in their preferred language, both in writing and via verbal discussion. The study was approved by the Western Institutional Review Board, Inc. (Protocol Number: 20190122).

### 2.3 Measures

The outcome measure was CHB medication adherence, assessed with the eight-item Morisky Medication Adherence Scale (MMAS-8) (26) at both baseline and 12-month follow-up assessment points. The MMAS-8 is an eight-item scale that has strong reliability and validity for measuring medication adherence among patients living with chronic conditions (27). Using MMAS-8, the participants in this study self-reported their CHB medication adherence by answering “yes” or “no” to seven questions and choosing one response option from a 5-item Likert response scale in the eighth question. The MMAS-8 score ranges from 0 to 8.

As a control variable, depression was assessed using the Patient Health Questionnaire-9 (PHQ-9), a widely used and validated self-report instrument for screening and measuring the severity of depression. The PHQ-9 consists of nine items assessing depressive symptoms over the prior 5 weeks, with total scores ranging from 0 to 27, where higher scores indicate greater depressive severity. Socioeconomic covariates, including participants’ age, gender, ethnicity, years lived in the US, marital status, education level, employment status, annual household income, and English-speaking proficiency, as well as health conditions and modifiable lifestyle behaviors, were included in the regression model on CHB medication adherence.

### 2.4 Statistical analysis

We compared intervention and control groups on several sociodemographic characteristics and modifiable lifestyle behaviors, using cross-tabulations and chi-square tests if the variables were categorical or t-tests if the variables were continuous. We then fitted an ordinary linear regression model to assess the intervention effects on CHB medical adherence at a 12-month follow-up assessment, controlling for sociodemographic and health-related characteristics. We conducted all statistical analyses in R (28). A *p*-value smaller than 0.05 was considered statistically significant.

## 3 Results

As shown in Table 1, among 129 Asian American adults who were on CHB medication and living in the Greater Philadelphia area and New York City, 52.7% were male, and 47.3% were female, with the mean age being 52.73. More than two-thirds (70.5%) of the participants were Chinese, with the remaining 29.5% being Vietnamese. In general, the socioeconomic status of the participants was low; about half of the participants reported lower than $20,000 annual household income, and the majority of them reported “high school or lower” education (72.9%) and “not at all/not well” English proficiency level.

**Table 1:** Sociodemographic characteristics of participants stratified by study group (*N* = 129).

Characteristic	Intervention (*N* = 85)	Control (*N* = 44)	Total (*N* = 129)	*p*-value
Participant source				
Existing cohort	44 (51.8%)	31 (70.5%)	75 (58.1%)	**0.04**
New recruitment	41 (48.2%)	13 (29.5%)	54 (41.9%)	
Age in years				
Mean (SD)	52.97 (12.47)	52.30 (13.75)	52.74 (12.87)	0.78
Range	20.00–85.00	24.00–87.00	20.00–87.00	
Gender				
Male	48 (56.5%)	20 (45.5%)	68 (52.7%)	0.24
Female	37 (43.5%)	24 (54.5%)	61 (47.3%)	
Ethnicity				
Chinese	57 (67.1%)	34 (77.3%)	91 (70.5%)	0.23
Vietnamese	28 (32.9%)	10 (22.7%)	38 (29.5%)	
Employment				
Employed	54 (63.5%)	31 (70.5%)	85 (65.9%)	0.42
Unemployed	5 (5.9%)	4 (9.1%)	9 (7.0%)	
Not in labor force	26 (30.6%)	9 (20.5%)	35 (27.1%)	
Annual household income				
≤$19,000	39 (45.9%)	25 (56.8%)	64 (49.6%)	0.24
≥$20,000	46 (54.1%)	19 (43.2%)	65 (50.4%)	
Education				
≤High school	56 (65.9%)	38 (86.4%)	94 (72.9%)	**0.01**
≥College	29 (34.1%)	6 (13.6%)	35 (27.1%)	
English proficiency				
Not at all/not well	62 (72.9%)	34 (77.3%)	96 (74.4%)	0.59
Well/very well	23 (27.1%)	10 (22.7%)	33 (25.6%)	
Years lived in the US				
<10 years	13 (16.0%)	5 (11.4%)	18 (14.4%)	0.48
≥10 years	68 (84.0%)	39 (88.6%)	107 (85.6%)	
Marital status				
Married	73 (85.9%)	37 (86.0%)	110 (85.9%)	0.98
Not married	12 (14.1%)	6 (14.0%)	18 (14.1%)	

Bold values indicate statistically significant results. *p < 0.05, **p < 0.01.

Participants’ modifiable lifestyle behaviors are described in Table 2. The mean scores of medication adherence at baseline and 12-month follow-up in the intervention group were significantly different from the scores of the control group (6.43 vs. 5.17, *p* = 0.004; 7.20 vs. 6.18, *p* < 0.001).

**Table 2:** Modifiable lifestyle behaviors of participants stratified by study arm (*N* = 129).

Lifestyle characteristic	Intervention (*N* = 85)	Control (*N* = 44)	Total (*N* = 129)	*p*-value
Had a regular physician				
No	4 (4.9%)	2 (5.1%)	6 (5.0%)	0.95
Yes	78 (95.1%)	37 (94.9%)	115 (95.0%)	
Had health insurance				
No	5 (5.9%)	7 (15.9%)	12 (9.3%)	0.06
Yes	80 (94.1%)	37 (84.1%)	117 (90.7%)	
Smoking				
Do not smoke	77 (90.6%)	40 (95.2%)	117 (92.1%)	0.36
Smoke	8 (9.4%)	2 (4.8%)	10 (7.9%)	
Alcohol consumption				
Do not drink	79 (94.0%)	37 (90.2%)	116 (92.8%)	0.44
Drink	5 (6.0%)	4 (9.8%)	9 (7.2%)	
Had heart diseases				
No	81 (95.3%)	42 (95.5%)	123 (95.3%)	0.97
Yes	4 (4.7%)	2 (4.5%)	6 (4.7%)	
Had hypertension				
No	68 (80.0%)	37 (84.1%)	105 (81.4%)	0.57
Yes	17 (20.0%)	7 (15.9%)	24 (18.6%)	
Had physical activities				
No	22 (26.2%)	14 (34.1%)	36 (28.8%)	0.36
Exercise	62 (73.8%)	27 (65.9%)	89 (71.2%)	
Had diabetes				
No	75 (88.2%)	37 (84.1%)	112 (86.8%)	0.51
Yes	10 (11.8%)	7 (15.9%)	17 (13.2%)	
Depression (PHQ9 score)				
Mean (SD)	4.42 (4.61)	5.57 (5.75)	4.81 (5.04)	0.22
Range	0.00–23.00	0.00–17.00	0.00–23.00	
Baseline medication adherence score				
Mean (SD)	6.43 (1.96)	5.18 (2.63)	6.01 (2.27)	**0.004**
Range	1.00–8.00	1.00–8.00	1.00–8.00	
12-month medication adherence score				
Mean (SD)	7.20 (1.21)	6.18 (1.77)	6.85 (1.50)	**<0.001**
Range	3.00–8.00	3.00–8.00	3.00–8.00	

Bold values indicate statistically significant results. *p < 0.05, **p < 0.01.

We then conducted multivariable linear regression analysis to examine the intervention effects on improving CHB medication adherence (Table 3). The overall regression was statistically significant (*R*^2^ = 0.61, *F* (22.79) = 5.70, *p* < 0.001). The results showed that the intervention group was significantly more likely to have higher CHB medication adherence than the control group (coefficient = 0.56, *p* = 0.04), with demographics, modifiable lifestyle behaviors, and baseline medication adherence score held constant, representing a meaningful increase on an 8-point adherence scale and is comparable in magnitude to effects reported in prior behavioral adherence interventions. A higher level of depressive symptoms significantly predicted poor CHB medication adherence (coefficient = −0.10, *p* = 0.003). Better medication adherence at baseline was associated with better medication adherence at 12-month follow-up assessment (coefficient = 0.22, *p* = 0.005). Other socio-demographic, health-related, and comorbidity factors were not significantly associated with the outcome variable in the regression model (*p* > 0.05).

**Table 3:** Ordinary linear regression results on the medication adherence score at 12-month follow-up assessment (*N* = 129).

Independent variables	Coefficients (95% CI)	*t* values	*p* values
Intervention group (ref: control group)	0.56 (−1.10, −0.02)	−2.09	0.04*
Depression (PHQ-9) at baseline	−0.10 (−0.17, −0.04)	−3.05	0.003**
Medication Adherence Score (MMAS-8) at baseline survey	0.22 (0.07, 0.37)	2.87	0.005**
New participant (ref: existing cohort)	−0.17 (−0.72, 0.39)	−0.59	0.56
Socio-demographic characteristics			
Age in years	0.01 (−0.01, 0.04)	0.86	0.39
Female (ref: male)	−0.32 (−0.84, 0.20)	−1.22	0.27
Vietnamese ethnicity (ref: Chinese ethnicity)	0.52 (−0.16, 1.20)	1.52	0.13
Married (ref: not married)	−0.64 (−1.35, 0.06)	−1.82	0.07
Unemployment (ref: employed)	−0.38 (−1.21, 0.46)	−0.90	0.37
Not in labor force (ref: employed)	0.15 (−0.50, 0.79)	0.46	0.65
Annual household income > = $20,000 (ref: income <= $19,000)	0.14 (−0.39, 0.68)	0.54	0.59
Education > = college (ref: <= high school education)	0.47 (−0.18, 1.12)	1.43	0.15
Lived in the US > = 10 years (ref: lived in the US <10 years)	0.21 (−0.52, 0.94)	0.57	0.57
English proficiency well/very well (ref: not at all/not well)	−0.07 (−0.78, 0.64)	−0.19	0.84
Healthcare access			
Had a regular physician (ref: did not have regular physician)	−0.29 (−1.64, 1.05)	−0.43	0.66
Had health insurance (ref: did not have health insurance)	0.54 (−0.52, 1.62)	1.02	0.31
Lifestyle behaviors and chronic comorbidity			
Smoking (ref: did not smoke)	−0.68 (−1.66, 0.31)	−1.37	0.17
Drinking (ref: did not drink)	0.28 (−0.62, 1.18)	0.62	0.53
Had physical activities (ref: did not do physical activities)	0.22 (−0.34, 0.78)	0.77	0.44
Had hypertension (ref: did not have hypertension)	0.20 (−0.43, 0.83)	0.63	0.53
Had heart diseases (ref: did not have heart diseases)	−0.62 (−1.72, 0.48)	−1.12	0.26
Had diabetes (ref: did not have diabetes)	0.16 (−0.54, 0.86)	0.45	0.66

*R*^2^ = 0.61, *F* (22.79) = 5.70, *p* < 0.01.

*p < 0.05; **p < 0.01.

CI, confidence interval; MMAS-8, eight-item Morisky Medication Adherence Scale. Bold values indicate statistically significant results. *p < 0.05, **p < 0.01.

## 4 Discussion

Long-term and adequate adherence to laboratory monitoring and antiviral treatment is crucial in reducing the risk of HCC, end-stage liver disease, and other hepatitis-related sequelae among individuals with CHB (29, 30). It is estimated that inadequate monitoring and care contribute to poor outcomes, as well as to increased CHB-associated healthcare costs and significant social burden (31). Therefore, helping patients overcome the multilevel barriers they face in accessing and adhering to treatment over the long term is crucial in reducing liver disease and liver cancer burden.

As demonstrated in this study, a community-based culturally appropriate intervention can significantly improve adherence to medication among Chinese and Vietnamese Americans with CHB in a 12-month period. To the best of our knowledge, this is the first study targeting improving CHB medication adherence among underserved Asian American patients in greater Philadelphia and New York City within a culturally tailored, community-based intervention context. We used randomized control trials to balance the characteristics of our participants, allowing us to attribute differences in outcome to the intervention (32). Our study revealed that a community-based, culturally appropriate intervention was critical in increasing medication adherence among CHB patients. Although MMAS-8 does not define strict clinical thresholds, a 0.56-point increase on an 8-point scale represents a nontrivial improvement in self-reported medication adherence. For example, a 2025 systematic review of mobile app interventions showed significant increases in MMAS-8 scores (mean difference ~0.57), indicating improved adherence across chronic conditions (33). In our study, for some participants, this magnitude of change (0.56-point increase) may correspond to movement across commonly used adherence categories (e.g., from low to medium adherence), suggesting potential clinical relevance at the population level. Per our findings, the intervention group had a higher mean medication adherence score than the control group and was more likely to have higher CHB medication adherence. The results indicate a significant intervention effect, similar to existing research on the effects of peer education in patients with CHB (34), in which the mean MMAS-B medication adherence score in the intervention group was significantly higher than in the control group (7.18 vs. 5.16, *p* < 0.05) (34).

Furthermore, we adopted a social-ecological perspective and CBPR approach to design and implement the multicomponent, language-congruent intervention. We engaged key stakeholders in all intervention design and implementation stages; stakeholders helped ensure that the study was culturally appropriate and that our intervention addressed the needs and concerns of participants. In addition, the bilingual patient navigators work closely with participants to address barriers to accessing and adhering to care for CHB. Some recent literature highlights similar participatory and community-engaged designs in health intervention research. For example, multilevel CBPR and participatory frameworks have been used to co-develop culturally tailored interventions that promote preventive health behaviors and advance health equity in diverse and marginalized communities, reinforcing the value of the collaborative approaches across clinical, public health, and community practices (35, 36). Aligning with recent CBPR-informed intervention studies, our study contributes to this growing literature by providing empirical evidence from Chinese and Asian American populations that collaborative, CBPR-guided interventions can effectively strengthen chronic hepatitis B (CHB) management and promoting health among racial and ethnic minority populations (37).

Moreover, we found that depressive symptoms were associated with medication adherence in this study, suggesting that mental health may be an important contextual factor in adherence-related interventions. Although causality cannot be inferred from this observational association, these findings highlight the potential value of considering mental health support as part of comprehensive strategies to promote medication adherence.

Our study is not without limitations. The self-reported measures used in this study may be subject to social desirability bias, and objective adherence indicators such as pharmacy refill records or clinical biomarkers were not available. Future studies incorporating objective adherence measures would provide a more comprehensive assessment of medication-taking behavior. It is also important to note that this study only recruited participants of Chinese or Vietnamese descent from the greater Philadelphia and metro New York City, which may limit the generalizability of the findings to Asian patients with CHB in other geographic regions. Future research should examine medication adherence and associated factors among other Asian American subgroups, such as Filipino Americans, and across different geographic regions to improve generalizability and achieve a more comprehensive understanding of adherence-related disparities. Moreover, MMAS-8 is an ordinal and bounded measure. Although it is frequently analyzed as a continuous outcome in adherence research, alternative modeling approaches may be explored in future work. Furthermore, the sample size was modest relative to the number of covariates included in the regression models. Although covariates were selected based on theoretical and empirical considerations to control for confounding, future research with larger samples may benefit from more parsimonious or penalized modeling approaches.

In conclusion, this study contributes to community-level health disparities research by demonstrating the potential benefits of culturally tailored multicomponent interventions for underserved Chinese and Vietnamese Americans living with CHB. Future research should focus on the implementation and dissemination of this evidence-based intervention among Asian subgroups who are disproportionately affected by CHB in order to help enhance our understanding of the best approaches to address health disparities among Asian American populations.

## Data Availability

The datasets presented in this article are not readily available because all data will be kept confidential to protect participants’ information. Requests to access the datasets should be directed to Grace X. Ma; grace.ma@temple.edu.
